# Leave it or fix it? How fixation of a small posterior malleolar fragment neutralizes rotational forces in trimalleolar fractures

**DOI:** 10.1007/s00402-021-03772-9

**Published:** 2021-01-28

**Authors:** Julia Evers, Maren Fischer, Michael Raschke, Oliver Riesenbeck, Alexander Milstrey, Dominic Gehweiler, Boyko Gueorguiev, Sabine Ochman

**Affiliations:** 1 University Hospital Muenster, Clinic for Trauma, Hand and Reconstructive Surgery, Albert-Schweitzer Campus 1, W1, 48149 Muenster, Germany; 2 AO Research Institute Davos, Clavadelerstrasse 8, 7270 Davos, Switzerland

**Keywords:** Trimalleolar fracture, Computed tomography, Ankle, Fracture, Posterior malleolar fragment, Positioning screw, Osteosynthesis

## Abstract

**Introduction:**

This study investigated the effects of a small posterior malleolar fragment (PMF), containing less than 25% articular surface area, on ankle joint stability via computed tomography (CT) scanning under full weight bearing in a human cadaveric ankle fracture model.

**Materials and methods:**

A trimalleolar fracture with a PMF of less than 25% articular surface area was created in 6 pairs of fresh-frozen human cadaveric lower legs. The specimens were randomized into 2 groups stabilized by internal fixation including a positioning screw for syndesmotic reconstruction. In Group I the PMF was addressed by direct screw osteosynthesis, whereas in Group II the fragment was not fixed. Six predefined distances within the ankle were measured under axial loading. CT scans of each specimen were performed in intact and fixated states in neutral position, dorsiflexion and plantar-flexion of the ankle.

**Results:**

In plantar-flexion, significant differences were detected between the groups with regard to rotational instability. Group II demonstrated a significantly increased inward rotation of the fibula compared with Group I.

No significant differences were detected between the groups for each one of the measured distances in any of the three foot positions.

**Conclusions:**

Additional reduction and fixation of a small PMF seems to neutralize rotational forces in the ankle more effectively than a sole syndesmotic screw. Clinically, this becomes relevant in certain phases of the gait cycle. Direct screw osteosynthesis of a small PMF stabilizes the ankle more effectively than a positioning screw.

## Introduction

The ideal treatment of a posterior malleolar fracture fragment (PMF) is an ongoing debate in the current literature. Not just the size of the fragment is important but also its shape in relation to the syndesmotic complex [1–4].

Anwar et al. were able to show that a direct screw osteosynthesis is beneficial versus an indirect screw osteosynthesis of PMFs [5]. Additionally, a meta-analysis and a clinical long-term study showed favorable results for the former [6, 7]. Gardner et al. reported that the stability achieved with a direct screw osteosynthesis of a PMF is superior to syndesmotic screw stabilization under torsional loading [8]. However, this type of loading loading is not the normal one an ankle has to bear during walking. This study was conducted to mimic loading of the ankle under full weight bearing during different phases of the gait cycle. It analyzed ankles with a trimalleolar fracture in three different positions of the foot – neutral position (NP), dorsiflexion (DF) and plantar-flexion (PF) – under axial loading to measure six predefined distances within the ankle that could point to signs of instability. The main difference between the two study groups was related to the treatment of the PMF. Following fixation of the medial and lateral malleolus and stabilizing the ankle with a syndesmotic screw, the PMF was treated with a direct screw osteosynthesis in Group I, while in Group II the PMF was not addressed. The treatment strategy for Group II represents the standard of care in most hospitals.

## Materials and methods

Ethical approval for the study was given by the institutional review board.

Six pairs of fresh-frozen ( – 20 °C) human cadaveric lower legs without any prior fractures were used in this study.

A range of motion (ROM) of at least 15° dorsiflexion and 25° plantar-flexion of the ankle was defined as necessary and tested in all ankles.

All specimens were thawed for 24 h at room temperature before preparation and testing. Amputation of each specimen was performed at the level of tibial tuberosity.

The specimens were assigned pairwise to two study groups with six specimen each (*n* = 6) and an equal number of right and left legs in each group.

Trimalleolar fractures (Supination-Eversion Fracture Stadium IV according to Lauge-Hansen classification or 44-B3.3 according to the AO/OTA classification) were created in all specimens. Starting with a standardized osteotomy of the distal fibula, a Weber B fracture was simulated with an additional dissection of the anterior syndesmosis. Then a fracture of the medial malleolus was created. Finally, a PMF categorized as type-1 (postero-lateral oblique type) according to Haraguchi et al. [9] and type-2 according to Bartoníček et al. [1], was set, involving less than 25% of the articular surface area with an angle of 20° to the bimalleolar axis. Form and size of the created PMF were verified with a CT scan.

Thereafter, the fractures were fixed with sequential open reduction internal fixation (ORIF) technique of the medial and lateral malleolus, including a positioning screw for syndesmotic reconstruction [10].

**Fig. 1:**
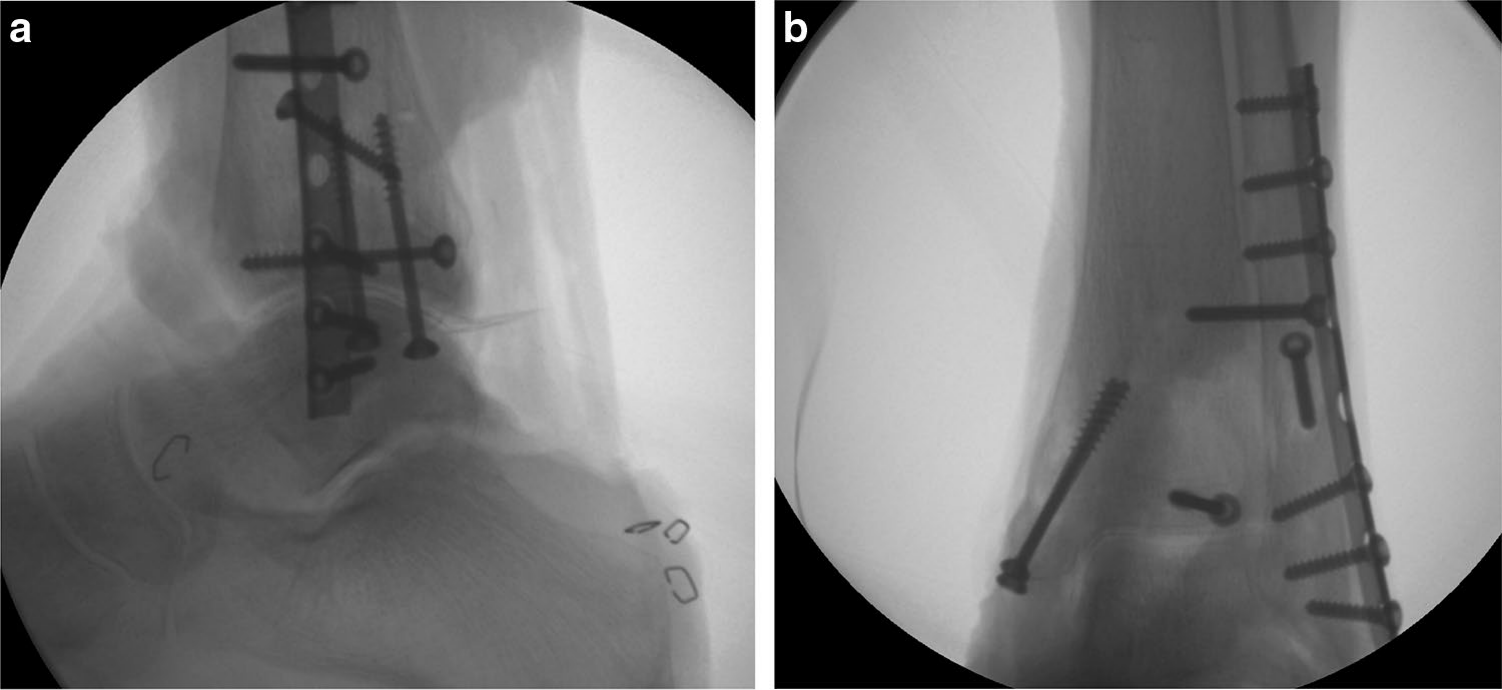
**a**, **b** Radiographs in two planes of a specimen after osteotomy and osteosynthesis with fixation of PMF (Group I)

**Fig. 2:**
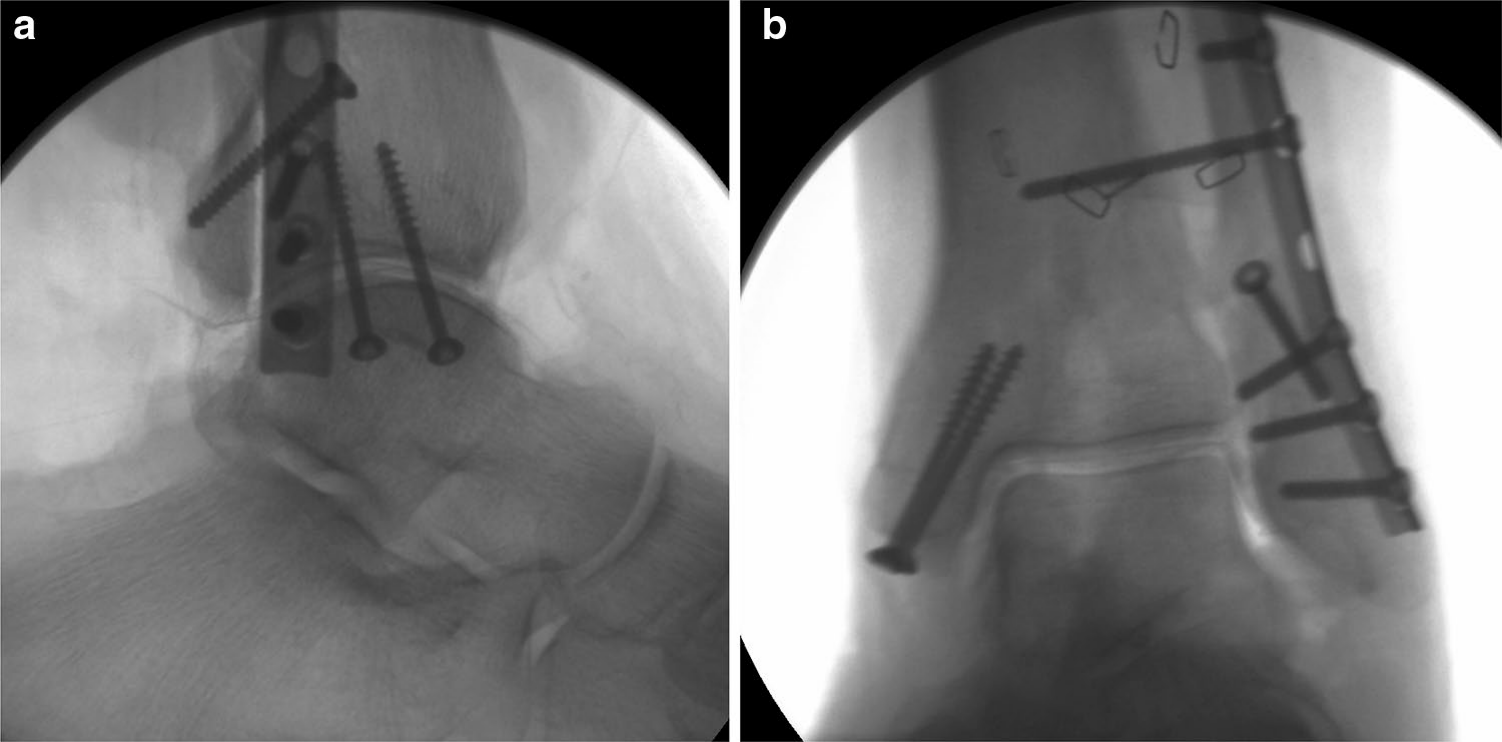
**a**, **b** Radiographs in two planes of a specimen after osteotomy and osteosynthesis without fixation of PMF (Group II)

In Group I, the PMF was fixed additionally with one partially threaded 4.0 mm lag-screw of appropriate length after direct open reduction (Fig. 1a,b), while the PMF of the specimens in Group II was treated conservatively (Fig. 2a,b). Reduction of all fractures was verified with fluoroscopic control (ARCADIS Varic, Siemens Healthcare GmbH, Erlangen, Germany).

**Fig. 3:**
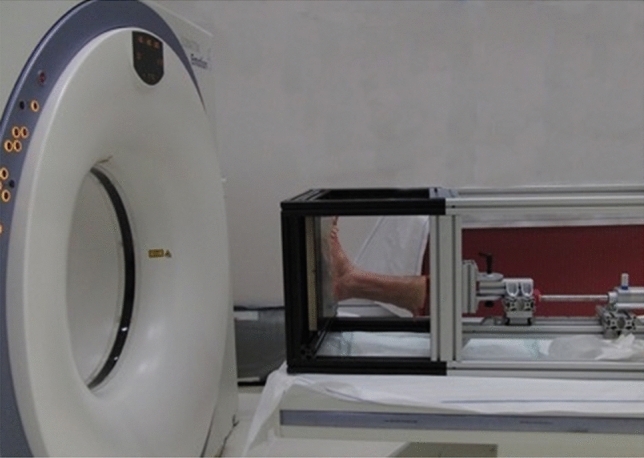
A specimen mounted for CT scanning under axial loading in neutral position in the air-pressure-controlled frame

In preparation for testing, the proximal 5 cm of the tibia and fibula were embedded in a polymethylmethacrylate (PMMA) block (Beracryl; Suter Kunststoffe AG, Fraubrunnen, Switzerland). The arches of the feet were stabilized by a moulded PMMA sole from plantar, customized individually for each foot. The specimens were mounted for testing in an air pressure-controlled frame (Fig. 3). Axial loading in NP was performed with vertically placed sole, for loading in DF and PF wooden wedge-shaped blocks were positioned under the PMMA sole.

Subsequently, weight-bearing CT scans of each specimen were obtained in NP, 15° DF and 25° PF of the ankle under 720 N static load in a Somatom Emotion CT scanner (Siemens Healthcare GmbH, Erlangen, Germany) with a 0.63 mm slice resolution. All measurements for every ankle were conducted in neutral position (NP), according to the neutral zero method.

Each specimen was loaded three times in intact state – in NP, DF and PF – and then three times following both fractured state and reconstructed state with fixation with the same three different positions of the foot.

The quality of reduction was verified using image processing software (OsiriX Lite, Bernex, Switzerland). Less than 2 mm step-off was considered a good reduction. Furthermore, the size of the PMF was determined with the same software and measured as percentage of the involved articular surface area.

Six distances were defined and measured in each specimen's state and foot position.

**Fig. 4:**
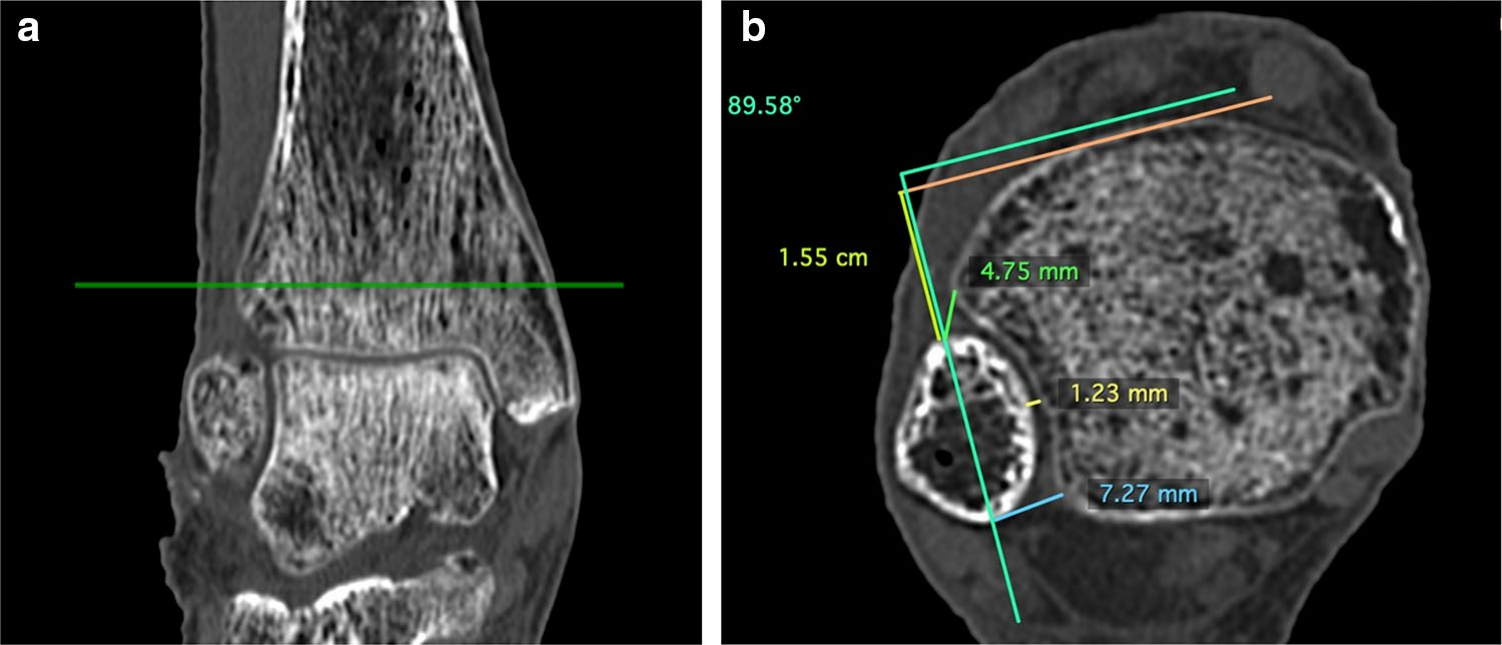
**a**, **b** CT scan of an ankle in neutral position. **a** visualisation of a reference line 1 cm above the joint line in the coronal plane. **b**  visualisation of the measures: Syn_post (7.27 mm), Syn_trans (1.23 mm), Syn_ant (4.75 mm), Rotation (89,58°) and Translation (1.55 cm). The tangential line just in front of the tibia is taken as a reference line for the measurements of the rotational angle and translation of the fibula [11]

**Fig. 5:**
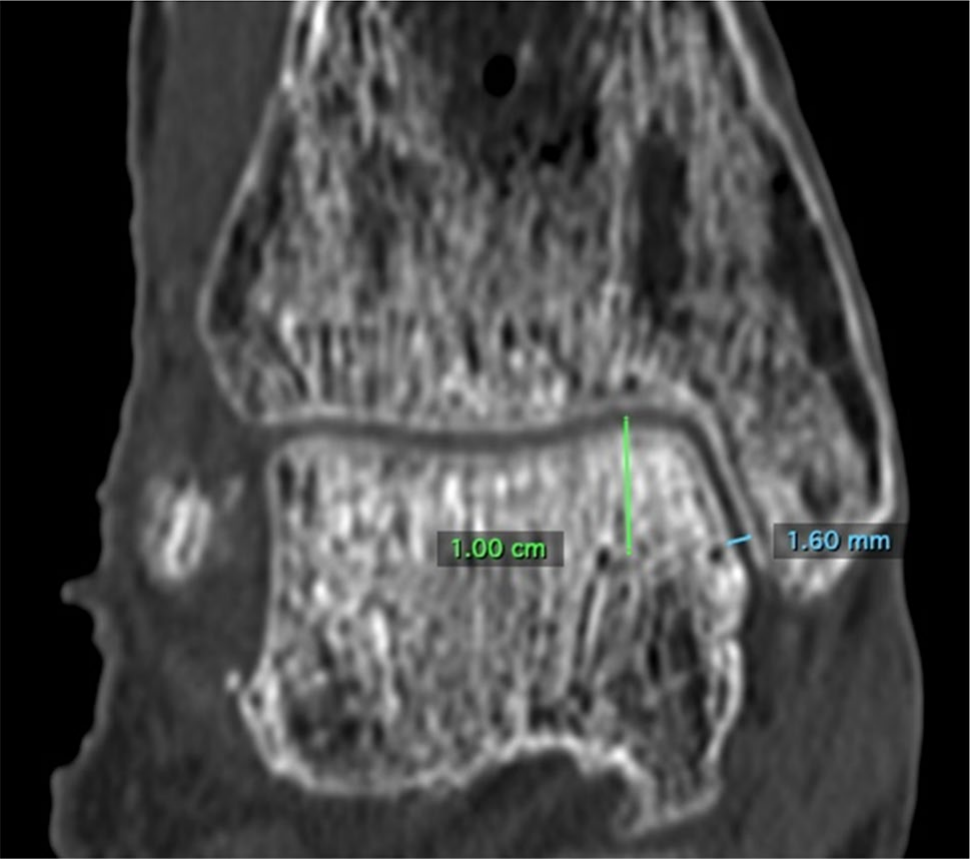
MSC measurement in an ankle in neutral position for the MCS 1 centimeter below the joint line

The osseous gap of the anterior syndesmosis (Syn_ant), the gap of the posterior syndesmosis (Syn_post) and the tibiofibular clear space (Syn_trans) were measured one centimeter above the joint line.Moreover, both the rotation of the fibula and its translation in relation to the tibia were quantified according to a method described by Zwipp [11] (Fig. 4). In addition, the medial clear space (MCS) was measured one centimeter below the joint line (Fig. 5) [12].

All measurements were carried out by three experienced foot and ankle surgeons. Each measurement was repeated 3 times to calculate its median and 1^st^/3^rd^ quartiles (Table 1).

**Table 1:** Median and 1st/3rd quartiles of the measures among all intact specimens in different foot positions

	Neutral position		Dorsiflexion		Plantar-flexion	
	Median	1^st^/3^rd^ quartile	Median	1^st^/3^rd^ quartile	Median	1^st^/3^rd^ quartile
Syn_ant [mm]	2.88	2.2/3.4	3.3	2.7/3.9	2.43	1.8/3.1
Syn_post [mm]	6.87	6.4/7.4	7.04	6.2/7.5	6.46	5.7/6.7
Syn_trans [mm]	1.76	1.5/2.6	1.94	1.6/2.8	1.35	1.1/2.2
MCS [mm]	2.17	1.8/2.4	2.07	1.6/2.3	2.47	2/2.8
Rotation [°]	89.04	87.4/91.8	89.55	87.2/92.5	89.73	85.6/92.1
Translation [mm]	11.93	10.4/14.1	12.6	10.9/14.8	12.39	10.2/13.4

All data was collected under loading in both the state and the fractured state after osteosynthesis of the specimens.

For each separate measure, the changes between the intact state and the state after osteosynthesis were evaluated.

### Statistical analysis

All descriptive results are given in terms of median and 1^st^/3^rd^ quartile. The analysis was performed using SPSS software package (version 25, IBM, Armonk, New York, USA). The Wilcoxon Signed-Rank test and the Mann–Whitney test were used to detect significant differences between paired and unpaired groups of measures, respectively. The level of significance was set at *p*  > 0.05 for all statistical tests. Bonferroni correction was considered for multiple comparisons.

## Results

The absolute measures demonstrated slightly smaller values, especially with regard to MCS and Syn_Trans, when compared to previous CT studies under weight bearing (Table 1) [13].

Rotational changes of the fibulae in the intact ankles between NP, DF and PF showed a heterogenic array of values. Some fibulae demonstrated an inward rotation, some- an outward rotation, independent from the dorsi- or plantar-flexion of the foot. The median values reflect these findings, as there is no difference visible between the foot positions. A negative value represents an outward rotation, while a positive value – an inward rotation (Tables 2 and 3).

**Table 2:** Changes in the measures between intact ankles and their state after osteosynthesis in Group I

Specimens and positions	Syn_ant [mm]	Syn_trans [mm]	Syn_post [mm]	MCS [mm]	Rotation [°]	Translation [mm]
1R NP	1.38	2.16	1.6	– 0.02	0.71	– 0.1
1R DF	1.53	1.69	1.47	0.34	– 0.33	– 0.39
1R PF	0.77	2.15	1.73	0.17	1.78	– 1.11
2R NP	0.27	1	1.44	0.69	0.59	– 0.89
2R DF	– 0.23	1.34	1.11	0.31	– 0.92	– 0.95
2R PF	1.12	0.51	1.31	1	– 0.46	0.37
4L NP	0.38	1.17	1.32	– 0.14	0.74	– 0.56
4L DF	0.23	0.69	0.79	0.23	1.54	– 0.56
4L PF	0.62	0.96	1.03	0.48	– 1.34	1.14
5R NP	0.13	1.33	1.63	0.06	1.54	– 0.82
5R DF	0.07	0.74	1.3	– 0.03	0.24	– 1.39
5R PF	0.6	1.1	1.76	0.33	3.08	– 0.76
7L NP	0.18	0.36	0.45	0.19	0.06	– 0.96
7L DF	– 0.05	0.8	0.46	0.35	– 0.61	– 0.7
7L PF	0.21	1.09	0.5	0.81	0.86	– 0.51
8R NP	1.08	0.03	1.24	0.34	– 0.71	– 0.04
8R DF	0.73	0.47	1.01	0.32	1.61	– 0.76
8R PF	0.81	– 0.53	0.84	0.07	– 1.38	0.03

**Table 3:** Changes in the measurements between intact ankles and their state after osteosynthesis in Group II

Specimen and position	Syn_ant [mm]	Syn_trans [mm]	Syn_post [mm]	MCS [mm]	Rotation [°]	Translation [mm]
1L NP	0.1	– 0.92	0.85	0.23	– 0.72	– 0.76
1L DF	– 1.02	– 0.3	0.34	0.13	1.39	– 2.16
1L PF	– 0.94	0.42	1.41	0.27	2.07	– 2.6
2L NP	1.88	– 1.55	0.4	0.23	– 3.74	1.63
2L DF	0.86	– 0.29	0.66	0.31	– 3.56	0.46
2L PF	2.01	0.8	0.26	0.14	– 5.03	1.09
4R NP	0.92	0.85	– 1.19	0.31	– 0.62	0.36
4R DF	0.68	1.09	1.08	0,23	0.16	– 0.06
4R PF	– 0.62	0.7	– 0.27	0.35	2.57	– 0.48
5L NP	– 0.26	– 0.18	– 0.47	0.05	1.77	– 0.44
5L DF	– 0.86	– 0.19	– 0.38	0.08	2.18	– 0.7
5L PF	– 0.62	0.7	– 0.27	0.35	2.57	– 0.48
7R NP	– 0.59	– 0.47	0.62	0.19	4.32	– 0.11
7R DF	– 0.1	– 0.15	0.56	0.05	6.2	– 0.87
7R PF	0.09	– 0.31	0.97	0.45	3.7	0.3
8L NP	– 0.79	– 1.46	– 0.37	0.14	4.56	– 0.59
8L DF	– 0.88	– 0.58	– 0.37	– 0.01	3.24	– 0.49
8L PF	0.04	– 0.77	0.21	0.38	3.57	0.29

Analyzing the changes in the measures comparing Group I and Group II showed differences, especially for rotation.

Regarding the rotational movement of the distal fibula, a significantly bigger change in inward rotation was registered in Group II (median 3.5°) versus Group I (median 1.5°) under PF of the ankle (*p* = 0.003).

In NP of the ankle, no significant changes (*p* = 0.636) were detected, whereas in DF we could see a trend to significant difference (*p* = 0.053). Additionally, in NP and DF the fibula tended to show an increased inward rotation in Group II compared to Group I.

Looking at the measures related to the anterior or posterior syndesmosis (Syn_ant, Syn_post) or tibiofibular clear space (Syn_trans), no distinctive changes were found.

Syn_ant showed a median change of 0.65 mm (± 0.11 mm standard deviation) in all examined positions of the ankle, whereas the Syn_trans median change in those positions was 0.92 mm (± 0.29 mm) - as measured between the intact state and the fractured state after osteosynthesis.

Syn_post resulted in median change of 0.81 mm (± 0.25 mm).

**Fig. 6:**
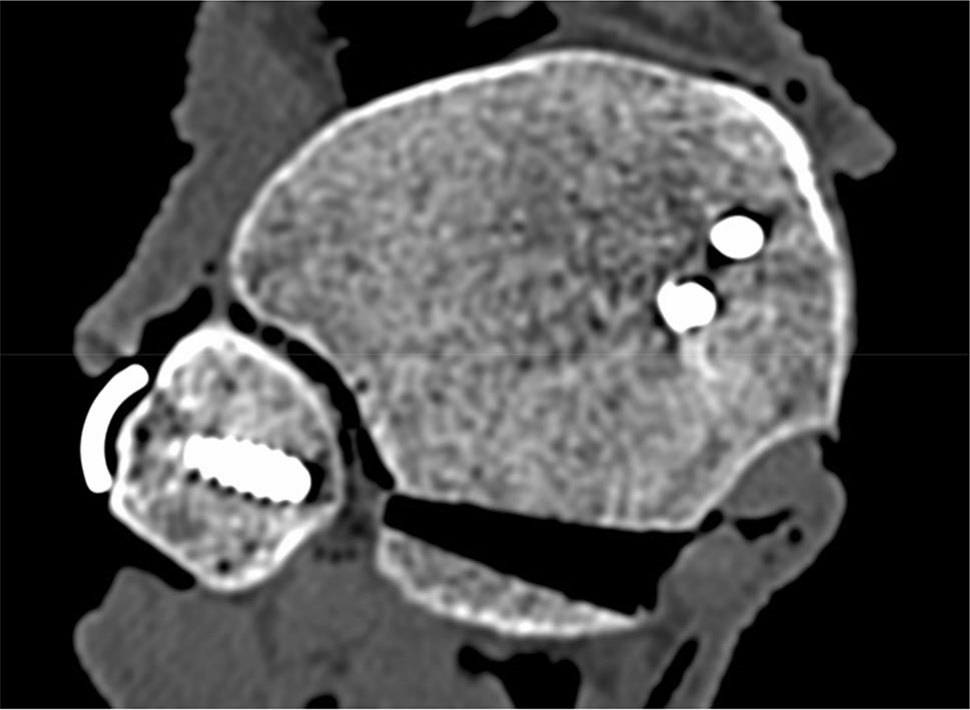
Axial CT slice of a specimen in neutral position under axial loading. With dislocation of the posterior fracture fragment in Group II, the distances for Syn_trans and Syn_post tended to be smaller than in Group I

Specifically, for changes in Syn_trans and Syn_post in Group II, these distances tended to be smaller without fixation of the PMF due to an instability of the fracture fragment (Fig. 6).

The differences of these measures between Group I and Group II were as small or only slightly larger than the CT slice resolution and therefore not representative.

MCS also demonstrated no significant no significant differences between the two groups, with changes of approximately, were approximately 0.2 mm and therefore also below CT’s slice resolution.

During the examination of translational movements at the distal fibula the changes in the measures were approximately 0.7 mm.

## Discussion

For many years, the PMF was treated depending on its size, according to the recommendations by Nelson and Jensen [14].

Further studies of the ankle demonstrated the complexity of the joint movement [15] allowing only little alterations in a trimalleolar ankle fracture with rates for arthrosis as high as 34% [16]. Therefore, the idea arose that not only the size of a PMF mattered but rather its configuration and its linkage to other anatomical structures [1–4].

To mimic the actual standard of care, we reconstructed our trimalleolar fractures according to the AO standards and therefore implanted a positioning screw to address the instability of the syndesmosis additionally. We applied axial loading in three different foot positions to mimic the maximal load to the ankle joint in certain key moments of the gait cycle [17]. Since torsional moments have already been proven to stress the restoration of syndesmotic stability, we refrained from their application [8].

Inman could show that the talus does a rotational movement while changing from DF into of the foot. While the axis of the talus is oriented to the medial part of the ankle in PF, its orientation changes to the lateral direction of the ankle in DF [15].

Different examinations analyzed the motion of the distal syndesmosis during the stance phase of the gait cycle. From heel strike to mid-stance, in these studies the fibula everted, externally rotated relative to the tibia, and then moved to the reverse direction from mid-stance to toe-off [4, 18]. In our study, we aimed to mimic loading of an ankle under full weight bearing in different phases of the gait cycle.

Similarly to other biomechanical research, we have to accept some weaknesses because our study design can only reflect the actual in vivo conditions to a limited extend. Some inherent limitations of the human cadaveric studies are related to poor bone quality due to advanced age of the donors and the thawing process. Furthermore, we did not simulate the muscle forces acting at the lower legs.

To eliminate differences between the feet, we only compared the intact state of an ankle under axial loading in NP, DF and PF to its reconstructed state after osteosynthesis after osteosynthesis under the exactly same conditions. Our measures in DF and PF did not reflect the normal range of motion of a young healthy individual, but these measures were obtainable in every specimen of our study.

Our median values were equivalent when compared with current clinical weight-bearing CT studies [13]. This verified the clinical relevance of our simulated weight-bearing model.

Previous research demonstrated that weight-bearing CT scans are comparable to non-weight-bearing CT scans of the ankle with a plantigrade foot referring to translational and rotational movements of the distal fibula related to [13, 19]. Examining the standard measurements in the ankle related to the MCS and the tibiofibular distances demonstrated that weight bearing had no effect on the tibiofibular joint congruency, but on MCS, which tended to be smaller under weight bearing when compared with CT scanning slices without axial loading [13].

Lepojärvi et al. demonstrated in a weight-bearing CT study that in NP of the loaded ankle, the fibula was located anteriorly in the tibial incisura in 88% of the subjects. When the ankle was rotated, median anteroposterior motion was 1.5 mm and median rotation of the fibula was 3°. Between internal and external rotation there was no significant change in tibiofibular clear space [20].

Chao et al. showed that the posterior syndesmosis absorbs part of the weight-bearing load during the stance phase of the gait cycle, while the load on the posterior part of the distal tibia is increased when moving the ankle from DF to PF [21].

Fitzpatrick et al. demonstrated in a CT study of human cadaver specimens with weight bearing in NP that the overall anterior–posterior reduction of the syndesmosis was generally unaffected by a posterior malleolus fracture except the situation with malreduction of a large fragment. The medical–lateral syndesmotic reduction was affected by the conditions of the posterior malleolus fixation, with malreduction of the posterior malleolus leading to syndesmotic malreduction. Rotational instability was not analyzed [22].

In contrast, NP we exposed our specimens to axial loading in DF and PF and observed an increased inward rotation of the fibula during PF in those ankles, where the PMF was not stabilized by a screw osteosynthesis (Group II). With the use of a syndesmotic screw alone, the rotational instability of the fibula in relation to the tibia could not be restored.

Our study showed distinct fibular movement with missing reduction of a small PMF.

Although only axial and no torsional loading was applied during the CT scanning, rotational instability was could be detected, which could be explained by the shape of the talus and its inward directed pseudorotation during PF [16], and the increased loading of the PMF during PF [10, 21].

The syndesmotic screw seems to be too weak to compensate the torsional moments which apply especially in plantar-flexion in the ankle joint.

Therefore, an osteosynthesis of the small PMF stabilizes the ankle and neutralizes acting torsional moments.
